# *qAG2.1* Is Associated with Anaerobic Germination Tolerance in Rice Seeds: Evidence from Haplotype Analysis and Marker-Assisted Breeding

**DOI:** 10.3390/plants15050821

**Published:** 2026-03-07

**Authors:** Vijay Kumar Reddy Challa, Siddharth Panda, Annamalai Anandan, Sharat Kumar Pradhan, Aruna Yelemele Raghavendra Rao, Bhojaraja Naik Keshava

**Affiliations:** 1 Crop Improvement Division, Indian Council of Agricultural Research (ICAR)-National Rice Research Institute (NRRI), Cuttack 753006, India; 2 Department of Plant Breeding and Genetics, Odisha University of Agriculture & Technology, Bhubaneswar 751003, India; 3 ICAR-National Institute of Seed Science and Technology, Regional Station, GKVK Campus, Bengaluru 560065, India; 4 Indian Council of Agricultural Research, Krishi Bhavan, New Delhi 110001, India

**Keywords:** anaerobic germination, GWAS, MABC, haplotype, CR Dhan 801, *qAG2.1*, *Sub1*

## Abstract

Anaerobic germination tolerance (AGT) is a critical adaptive trait for rice establishment in flood-prone environments and direct-seeded systems. Here, we identified and validated the quantitative trait locus *qAG2.1* for AGT and introgressed it into the elite lowland rice variety CR Dhan 801 through marker-assisted backcross breeding. The introgressed lines exhibited significantly improved germination under anaerobic conditions, demonstrating the effectiveness of *qAG2.1* in a high-yielding genetic background. While CR Dhan 801 showed a low anaerobic germination percentage (17.6%), the donor ARC10424 exhibited 82.6%, and the best-performing introgressed line (22009-3) achieved 49.2%. Importantly, the improved lines maintained agronomic performance comparable to CR Dhan 801 under non-stress conditions, indicating minimal yield penalty. To gain mechanistic insight, the *qAG2.1* interval was dissected in silico to prioritise candidate genes putatively associated with AGT. This analysis highlighted genes linked to ethylene biosynthesis and signalling (e.g., *OsACO3*, *OsERF109*), abscisic acid biosynthesis (*OsNCED1*), gibberellin homeostasis (*OsGA2ox9*), trehalose metabolism (*OsTPS5*, *OsTPP1*), detoxification of anaerobic by-products (*OsALDH2A*), and water transport (*OsPIP1;3*). Collectively, these results validate *qAG2.1* as a further deployable locus for improving anaerobic germination in elite rice backgrounds and provide a set of putative candidate genes for future functional characterisation.

## 1. Introduction

Anaerobic germination tolerance (AGT) is a crucial adaptive trait for rice (*Oryza sativa* L.), particularly in regions prone to flooding and those adopting direct-seeding systems. Direct-seeding has gained popularity in rice cultivation due to its cost-effectiveness, faster crop establishment, and enhanced early-stage growth compared to traditional transplanting methods [1,2]. However, the success of direct-seeding systems is largely dependent on the ability of rice varieties to germinate and establish growth under low-oxygen conditions, a significant challenge in areas experiencing intermittent flooding [3]. Under submerged conditions, rice seeds face the need to elongate shoot tissues to escape floodwaters, a process that is essential for successful germination and seedling survival [4,5]. Improving AGT is therefore paramount for ensuring stable and consistent rice yields in flood-prone areas [6,7].

Earlier studies have identified key genetic loci associated with AGT, focusing on traits such as shoot length, root elongation, and biomass accumulation [7,8]. Advanced mapping techniques, including interval mapping (IM) and composite interval mapping (ICIM), have proven instrumental in uncovering quantitative trait loci (QTLs) linked to AGT [4,9]. Notable among these QTLs are *qAG9.1*, *qAG9.2* and *qAG11* [10,11]. Additionally, genes involved in ethylene biosynthesis, such as *OsERF109* and *OsACO3*, were reported to play key roles in regulating the plant’s response to submergence stress [12,13,14]. Ethylene, a plant hormone, triggers the rapid elongation of stems and leaves, which is essential for rice to escape from floodwaters [13,15]. Variations in the expression of genes like Os*ERF109* have been shown to influence germination success, with different haplotypes directly affecting AGT [16]. Similarly, Os*ACO3*, an enzyme involved in ethylene biosynthesis, modulates ethylene production during submergence, enhancing seedling development and anaerobic tolerance [12]. In addition to ethylene-related genes, several other factors, such as abscisic acid (ABA) and gibberellins (GAs), are critical for seed germination and seedling growth under anaerobic stress. ABA, a stress hormone, regulates seed dormancy and inhibits germination under unfavourable conditions. However, suppression of ABA biosynthesis, particularly through genes such as *OsNCED1*, promotes successful anaerobic germination [17]. Conversely, GA-related genes like *OsGA2ox9* help modulate gibberellin levels, which in turn enhance starch hydrolysis during germination, a key process for seedling emergence under low-oxygen conditions [18,19]. Furthermore, trehalose metabolism plays a significant role in rice under anaerobic conditions. Genes, such as *TPS5* and *TPP1*, which are involved in the synthesis and breakdown of trehalose, a sugar that provides energy for seed germination under oxygen-limited environments, are critical for supporting rice seedlings under submerged conditions [20,21,22,23,24]. Another critical aspect of AGT is the detoxification of harmful by-products produced under anaerobic stress. Aldehyde dehydrogenases (ALDHs), such as *OsALDH2A*, play a crucial role in detoxifying by-products like acetaldehyde, which accumulate during anaerobic stress. The activity of *OsALDH2A* is upregulated in rice varieties capable of successful anaerobic germination, aiding in detoxification and improving seedling survival under submerged conditions [3,25,26]. In addition, aquaporins, specifically *OsPIP1;3*, regulate water uptake during seed germination and are essential for optimal seedling emergence, especially under hypoxic conditions [27].

In the recent past, significant advancements have been made in identifying QTLs associated with AGT. However, the dissection of the QTL, putative gene associations, and haplotyping have not been reported before. By integrating functional genomics, QTL analysis, and haplotype characterisation, this study explores the genetic foundations of AGT in rice. Haplotyping the identified putative genes and using the non-synonymous SNPs (nsSNPs) to group the population further confirms the efficacy of the gene, and a successful haplotype would enable further genomic selection [28,29,30,31]. The current report sheds light on the QTL *qAG2.1* by dissecting the genomic region for putative candidate genes, haplotyping, and introgressing this QTL in the genetic framework of CR Dhan 801, a widely cultivated lowland rice variety known for its multiple stress tolerances [32]. This variety is reported to contain the Sub1 gene for submergence tolerance, and *qDTY1.1*, *qDTY2.1* and *qDTY3.1* yield QTLs for drought tolerance [33]. The aim of this research was to introduce AGT-regulating loci in the background of CR Dhan 801, supplementing its existing abiotic stress loci. Hence, this study reports phenotyping and genotyping of the F_2:3_ mapping panel to identify QTLs for AGT, followed by in silico dissection of QTL intervals to prioritise candidate genes, evaluation/haplotyping analysis in the Bengal and Assam Aus Panel (BAAP) through haplotype-based analyses, and simultaneously introgression into the elite rice variety CR Dhan 801 for phenotypic validation. By phenotyping the QTL in the background of CR Dhan 801, we demonstrate that the introgression of *qAG2.1* significantly enhances AGT, crop establishment, and yield under both normal and anaerobic conditions. These findings underscore the potential of AG-tolerant rice varieties to bolster food security, particularly in flood-prone areas. Additionally, the results offer valuable insights for future breeding programs aimed at developing high-yielding, submergence-tolerant rice varieties, ultimately contributing to sustainable agricultural productivity in regions that are vulnerable to intermittent flooding.

## 2. Results

### 2.1. Pheno-Genotyping and Identification of QTL in the Mapping Panel for Anaerobic Germination Tolerance

**Figure 1:**
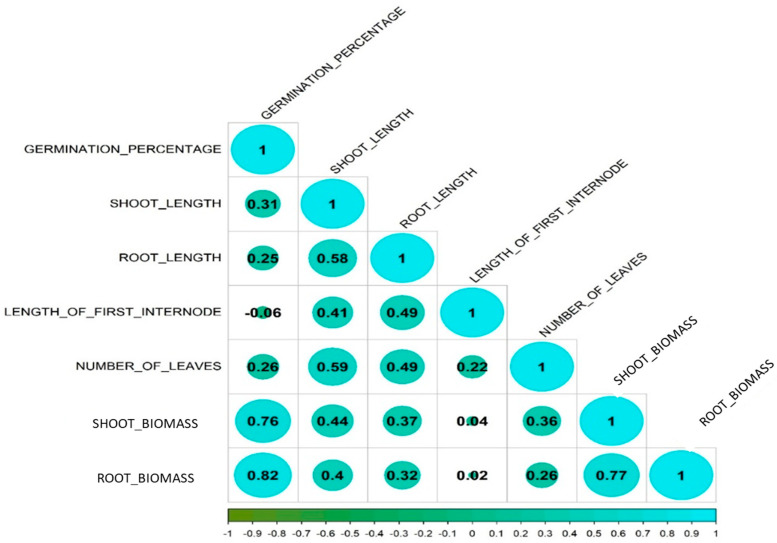
Estimates of Pearson correlation for traits studied in F_2:3_ mapping population under anaerobic stress conditions for the following traits: germination percentage (%), length of first internode (cm), shoot length (cm), number of leaves, root length (cm), shoot dry biomass (g), and root dry biomass (g). Colour (teal, positive correlation; green, negative correlation) intensity and the size of the circle are proportional to the correlation.

The phenotyping was done for the 172 F_2:3_ under anaerobic stress conditions for germination percentage, length of the first internode, shoot length, number of leaves, root length, shoot dry biomass, and root dry biomass under anaerobic conditions (Supplementary Table S1). Significant variations were observed across all traits; anaerobic germination percentage ranged from zero to 94.44%. Four lines had no germinated seedlings emerging above the water surface (were considered to be zero), while three lines had more than 90% of plants emerging above the water, i.e., >64 plants out of the total 72. The mapping population displayed large variations in both shoot and root traits under stress, where the root length was in the range of 3.5–18.50 cm, the length of the first internode ranged from 1.19 cm to 8.89 cm, and the shoot length ranged from 14.4 cm to 34.41 cm (Table 1). Statistical analyses revealed a higher phenotypic coefficient of variation (PCV) compared to the genotypic coefficient of variation (GCV) for all the traits, suggesting a notable influence of environmental factors on phenotype expression. Across traits, skewness values were close to zero to moderately negative (−2.18 to 0.31), indicating near-symmetric to mildly left-skewed distributions. Kurtosis values were mostly low to moderately positive (−1.30 to 7.82), suggesting distributions range from slightly platykurtic to moderately leptokurtic, without extreme heavy tails except for shoot length (kurtosis = 7.82). The correlation analysis elucidated the relationships among the studied traits under anaerobic growth conditions (Figure 1). Notably, anaerobic germination percentage displayed positive associations with shoot length (0.31), root length (0.25), number of leaves (0.26), shoot dry biomass (0.76), and root dry biomass (0.85). Conversely, shoot length exhibited positive correlations with root length (0.58), length of first internode (0.41), number of leaves (0.59), shoot dry biomass (0.44), and root dry biomass (0.40). Root length demonstrated positive correlations with all the traits.

**Figure 2:**
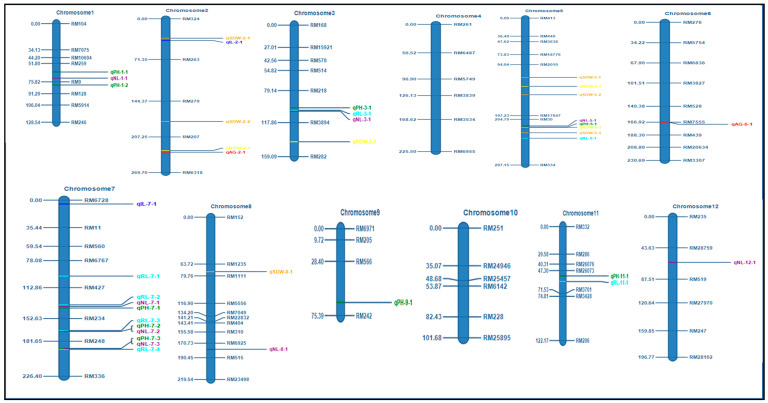
Genetic linkage map showing the positions of QTLs for seven traits related to anaerobic germination tolerance in the F_2:3_ population of ARC10424 developed in CR Dhan 801 background. QTLs were detected by interval mapping (IM) using QTL IciMapping software V. 4.1.

Parental polymorphism was studied using 702 SSR markers covering the 12 chromosomes. Of these, 81 (12.10%) SSR markers that showed clear, distinct polymorphic bands were further used to construct the linkage map and QTL analysis. The level of polymorphism ranged from 7.50 to 27.59% with an average of 13.25% for all the chromosomes (Supplementary Table S2). The linkage map was developed using these polymorphic markers, taking into account the Kosambi mapping function spanning across 2252.62 cM. The estimated map lengths (in cM) of the 12 linkage groups are furnished in Supplementary Table S3. This constructed linkage map served as a foundation for mapping the QTLs linked with the traits for AGT. The causative genomic regions were detected using interval mapping (IM) and inclusive composite interval mapping (ICIM) at an LOD threshold of 2.0. A total of 38 additive QTLs were detected by IM (Figure 2), and 33 additive QTLs were detected by ICIM for all AGT traits (Supplementary Figure S1). Details of IM and ICIM-QTLs with their flanking markers, position, LOD score, phenotypic variance, and additive effect are presented in Table 2.

Three QTLs associated with anaerobic germination were identified on chromosomes 2 and 6. On chromosome 2, *qAG2.1* was detected by both IM (LOD-2.85) and ICIM (LOD-3.06), explaining up to 12.4% and 5.45% of the phenotypic variance, respectively, while *qAG2.2* was identified only by ICIM with LOD 3.26 and PVE 11.69%. On chromosome 6, qAG6.1 showed consistent detection across methods with LOD 2.67 and 3.28, and PVE 1.67 and 3.4%. Additive effects indicated that favourable alleles were contributed by both parents, with chromosome 2 harbouring QTLs of relatively larger effect. For shoot length (SL), eight QTLs were mapped across chromosomes 1, 3, 5, 7, 9, and 11. A major QTL, *qSL5.1*, exhibited a high LOD value (16.71) and was consistently detected by both methods. Chromosome 7 represented a prominent QTL hotspot, where three tightly linked loci (*qSL7.1*, *qSL7.2*, and *qSL7.3*) displayed strong and consistent effects. Additional moderate-effect QTLs were detected on chromosomes 1, 3, 9, and 11. The additive estimates confirmed contributions from both parental lines, with chromosome 7 playing a predominant role in governing shoot length. Two QTLs controlling internode length (IL) were identified on chromosomes 2 and 7. *qIL2.1* explained 7.58% of the phenotypic variance with LOD 3.38, whereas *qIL7.1* accounted for 2.15% (LOD 2.87). Both loci exhibited moderate effects, suggesting that internode length is regulated primarily by minor QTLs with distributed genomic effects. Root length (RL) was controlled by multiple QTLs located on chromosomes 3, 5, 7, and 11. A prominent QTL cluster was observed on chromosome 7 (*qRL7.1*–*qRL7.4*), with LOD values ranging from 3.23 to 9.39, indicating this region as a major hotspot for root elongation. Additional moderate-effect QTLs were detected on chromosomes 3 (*qRL3.1*), 5 (*qRL5.1*), and 11 (*qRL11.1*).

### 2.2. In Silico Analysis of the qAG2.1 QTL Region to Identify and Validate Putative Candidates via Haplotype Diversity for AGT in the BAAP

Three QTLs were detected for anaerobic germination percentage (Table 3). Among these, *qAG2.1*, located on chromosome 2, was selected for candidate gene identification owing to its significant PVE. The *qAG2.1* region on chromosome 2, spanning from 24.42 Mb to 35.37 Mb, was annotated using the MSU Rice Genome Annotation Project (version 7), which harboured 1384 gene loci. Further analysis was carried out by excluding transposable elements, retrotransposons, and hypothetical proteins. Among these, 66 loci were found to be associated with metabolic pathways essential for seed germination under anaerobic conditions from their functional annotations and earlier reports. (Supplementary Table S4).

Among the 66 identified gene loci associated with *qAG2.1*, ten genes had sufficient sequence information along with nsSNPs in the BAAP, which were used in haplotype studies. The genes were AUXIN RESPONSE FACTOR 8-OsARF8 (LOC_Os02g41800), 9-CIS-EPOXYCAROTENOID DIOXYGENASE-*OsNCED1* (LOC_Os02g47510), ETHYLENE RESPONSE FACTOR 109-*OsERF109* (LOC_Os02g52670), ALDEHYDE DEHYDROGENASE 2A-*OsALDH2A* (LOC_Os02g49720), TREHALOSE-6-PHOSPHATE SYNTHASE 5-*OsTPS5* (LOC_Os02g54820), TREHALOSE-6-PHOSPHATE PHOSHPHATASE 1-*OsTPP1* (LOC_Os02g44230), PLASMA MEMBRANE INTRINSIC PROTEIN 1;3-*OsPIP1;3* (LOC_Os02g57720), NITRITE REDUCTASE 2-OsNIR2 (LOC_Os02g52730), GIBBERELLIN 2-OXIDASE 9-*OsGA2ox9* (LOC_Os02g41954), and AMINOCYCLOPROPANE-1-CARBOXYLIC ACID OXIDASE 3-*OsACO3* (LOC_Os02g53180). The available SNP information of these genes was accessed from the snpseek database, and the presence of nsSNPs (if any) was used to develop haplotype groups and highlight the effect of non-synonymous SNPs on the mean AGT of the corresponding group. The BAAP was phenotyped under anaerobic conditions for germination and has been reported in our previous work [3]. The following section reports such genes that were grouped and displayed significant differences in the BAAP via haplotype groups. The nsSNP information and haplotype sequence, along with their means and number of genotypes in each haplotype, are furnished in Supplementary Table S5.

**Figure 3:**
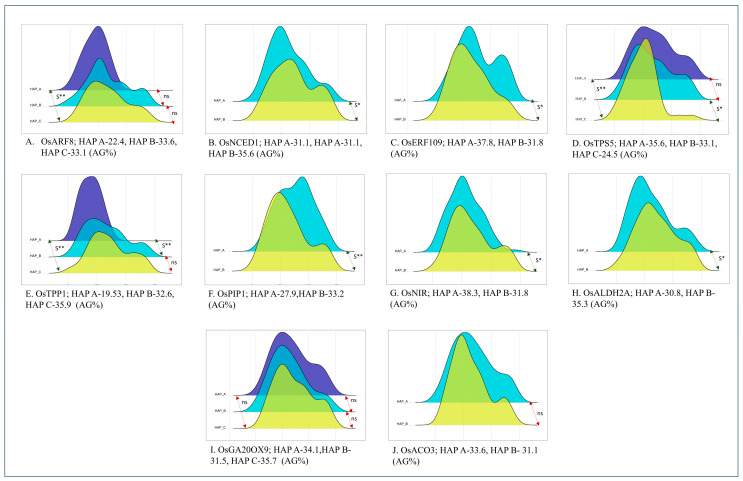
Ridgeline plot showing the variation among haplotypes and the significance between them across ten putative candidate genes identified in *qAG2.1* (significance level; *—*p* < 0.05, **—*p* < 0.01, S—significant, ns—nonsignificant).

The gene *OsARF8 (LOC_Os02g41800)* at 25,133,442 bp–25,136,111 bp plays a central role in controlling sensitivity to the plant hormone auxin. It has two nsSNPs positioned at 25,134,222 bp and 25,135,558 bp, displaying G/A and A/G polymorphism, respectively. These polymorphisms grouped the panel into three haplotype groups. The haplotype group HAP C with a G% of 33.1% was significantly different from HAP A with a G% of 22.4%. There was no significant difference between HAP A and HAP B, and HAP B and HAP C (Figure 3A). *OsNCED1 (LOC_Os02g47510),* which plays a crucial role in the ABA signalling pathway, was identified at 29,026,070 bp–29,028,259 bp with two nsSNPs positioned at 29,027,942 bp (T/C polymorphism) and 29,027,975 bp (A/G polymorphism), dividing the panel into two haplotype groups. HAP A and HAP B (116 genotypes) were significantly different from each other, with a mean G% of 31.1% and 35.6%, respectively (Figure 3B). The gene *OsERF109 (LOC_Os02g52670)* plays an important role in ethylene production and was identified between 32,204,392bp and 32,205,335 bp. It had one nsSNP at 2,205,001 bp with a T/A polymorphism, which divided the BAAP panel into two haplotypes. HAP A (G% of 37.8%) was found significantly different from HAP B (G% of 31.8%) (Figure 3C).

There were two genes that were found to be involved in trehalose metabolism, playing a crucial role in germination under anaerobic conditions. *OsTPS5* (LOC_Os02g54820), located between 33,569,263 bp and 33,574,120 bp, had nsSNPs at 33,570,743 bp (A/C polymorphism) and 33,571,460 bp (C/A polymorphism). They divided the entire BAAP panel into three haplotype groups: HAP A (G% of 35.6%) and HAP B (33.1%) were significantly superior to HAP C (G% of 24.5%). However, there was no significant difference between HAP A and HAP B (Figure 3D). The other gene *OsTPP1* (LOC_Os02g44230), located between 26,767,603 bp and 26,771,633 bp, had a total of two nsSNPs positioned at 26,768,242 bp (C/T polymorphism) and 26,768,540 bp (T/C polymorphism), dividing the BAAP panel into three distinct haplotype groups. The haplotype group HAP A, with a mean G% of 19.53%, was found significantly different from HAP B and HAP C, with a mean G% of 32.66% and 35.94%, respectively (Figure 3E).

The Aquaporin gene, *OsPIP1* (LOC_Os02g57720), stretching from 35,349,673 bp to 35,351,454 bp, plays a critical role in seed germination and the signal transduction of various hormones. This gene had one nsSNP, positioned at 35,349,952 bp (T/C polymorphism). This divided the panel into two haplotypes; HAP A with a G% of 27.9% was found to be significantly different from the superior haplotype HAP B with a G% of 33.2% (Figure 3F). *OsNIR2* (LOC_Os02g52730), found between 32,254,049 bp and 32,257,357 bp, plays a role in the nitrogen cycle and is involved in germination under hypoxic conditions. The BAAP sequence information identified five nsSNPs, grouping the panel into two haplotypes; HAP A with a G% of 38.3% was found superior and significantly different from HAP B with a G% of 31.8% (Figure 3G). *OsALDH2A* (LOC_Os02g49720), which was involved in detoxification of accumulated acetaldehyde under low oxygen conditions, was identified at 30,392,547 bp–30,396,656 bp with one nsSNP positioned at 30,395,020 bp (G/C polymorphism). The nsSNP divided the panel into two haplotypes: HAP A and HAP B, with a significant difference and a mean G% of 30.8% and 35.3%, respectively (Figure 3H).

It is noteworthy to mention that two hormone regulatory genes displayed no significant difference among the haplotypes despite having these nsSNPs. The first one was *OsGA2ox9* (LOC_Os02g41954), involved in GA metabolism (25,199,386 bp–25,203,742 bp) with two nsSNPs positioned at 25,203,252 bp (C/T polymorphism) and 25,203,261 bp (A/G polymorphism) (Figure 3I). Similarly, *OsACO3*–LOC_Os02g53180 (involved in ethylene homeostasis) was identified between 32,558,635 bp and 32,562,705 bp. This gene had seven nsSNPs. But the subgroups formed did not have any significant differences between them (Figure 3J).

### 2.3. Introgression of qAG2.1 in CR Dhan 801 Through Marker-Assisted Backcross

**Figure 4:**
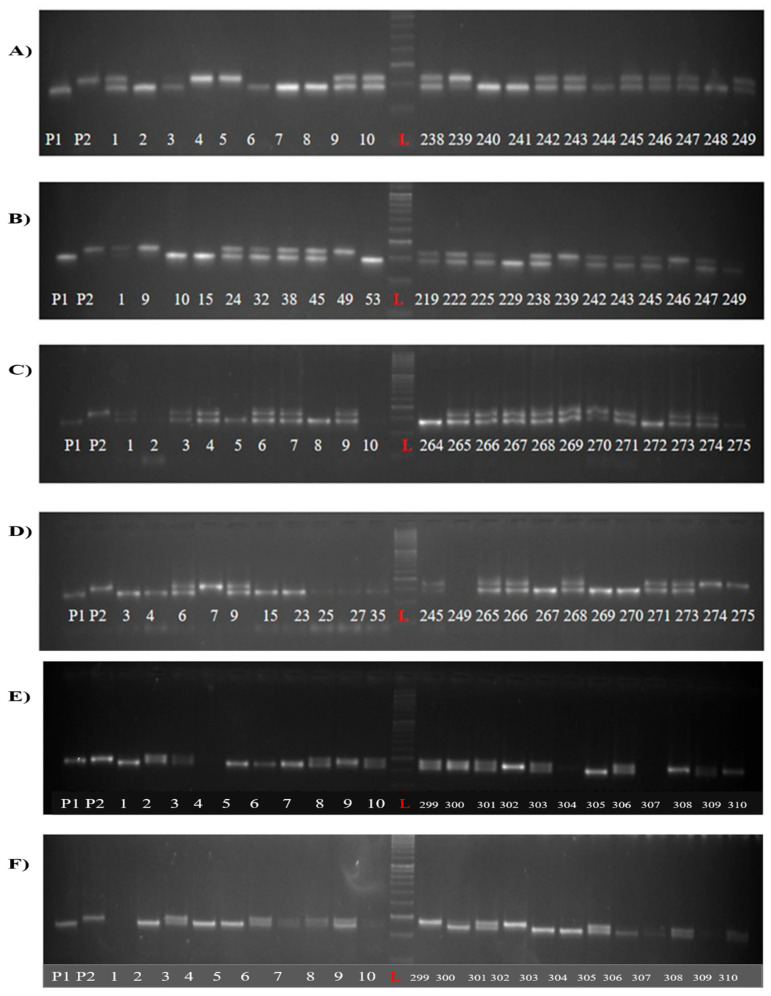
PCR amplification of markers linked to anaerobic germination QTL *qAG2.1* (RM 6318 left flanking marker (LFM), RM207 right flanking marker (RFM). (**A**) LFM of BC_1_F_1_, (**B**) RFM of BC_1_F_1_, (**C**) LFM of BC_2_F_1_, (**D**) RFM of BC_2_F_1_ (**E**) LFM of BC_3_F_1_, (**F**) RFM of BC_3_F_1_. P1—CR Dhan 801, P2—ARC10424, L—ladder–1000 bp.

To introgress the anaerobic germination tolerance QTL *qAG2.1* into the elite lowland variety CR Dhan 801, a marker-assisted backcrossing (MABC) scheme was implemented using ARC10424 as the donor parent. Polymorphism between ARC10424 and CR Dhan 801 was assessed at the *qAG2.1*-linked markers RM207 and RM6318 to enable foreground selection. Foreground selection for the *qAG2.1* QTL was conducted on all the BC_1_F_1_ plants, revealing 75 positives for *qAG2.1* (Figure 4A,B). Background screening of these lines for the genome of the recurrent parent was done using 81 SSR markers. The recurrent parent genome content in the 75 progenies ranged from 68.22% to 78.25%, with an average of 75.25%. The backcross derivative, 20,045, exhibiting 78.25% recurrent genome content, was selected to produce BC_2_F_1_ seeds. In the BC_2_F_1_ generation, 64 plants were positive for *qAG2.1* (Figure 4C,D). Background screening for the recovery of the recurrent parent genome in these positive plants revealed a range of 82.34% to 91.46%. Plant 21,055, which exhibited 91.46% recurrent genome recovery, was selected to produce BC_3_F_1_. The foreground genotyping results indicated 94 plants positive for *qAG2.1* (Figure 4E,F), while the background screening revealed a range of 88.21% to 94.25%. Plant 22011, which exhibited 94.25% recurrent genome recovery, was self-pollinated to produce BC_3_F_2_ seeds. All BC_3_F_2_ plants underwent foreground screening, and 156 plants were identified as homozygous for the *qAG2.1*.

**Figure 5:**
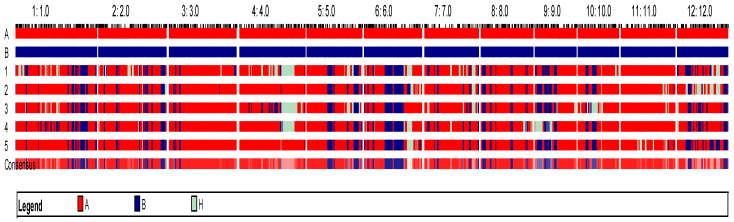
Graphical genotype (GGT) plot illustrating the recovery of the recipient parent genome in five BC_3_F_2_ introgressed lines. Red segments and blue segments represent homozygous regions from the recurrent parent (CR Dhan 801) and donor parent (ARC10424), respectively (A—CR Dhan 801, B—ARC10424, H—Heterozygous, 1—22004-3, 2—22004-16, 3—22004-17, 4—22011-18, 5—22011-20).

The recurrent parent CR Dhan 801 harbours the four important genes/QTLs for abiotic stress tolerance: Sub1, *qDTY1.1*, *qDTY2.1,* and *qDTY3.1* [32,33]. Thus, the 156 lines homozygous for *qAG2.1* were also checked for the presence of the above-mentioned four stress-regulating loci. The presence of Sub1 was determined using the Sub1BC2 [34] marker, for which 156 plants tested positive. The selected plants were further genotyped for the presence of drought tolerance QTLs *qDTY1.1* and *qDTY3.1* using the SSR markers RM431 [35] and RM416 [36], respectively. It is noteworthy to mention that the selected lines were devoid of *qDTY2.1*, as it co-localized with the target QTL, *qAG2.1*. Only five plants were confirmed to carry all targeted QTLs; these were 22004-3, 22004-16, 22004-17, 22011-18, and 22011-20. These lines were used to generate BC_3_F_3_ seeds, which were then evaluated for agro-morphological and yield-related traits under both normal and anaerobic stress conditions. The GGT plot in Figure 5 represents the five lines and their genome recovery in BC_3_F_1._

### 2.4. Evaluation of Agronomic and Yield Traits in MABC-Derived Lines Under Normal and Stress Conditions

**Figure 6:**
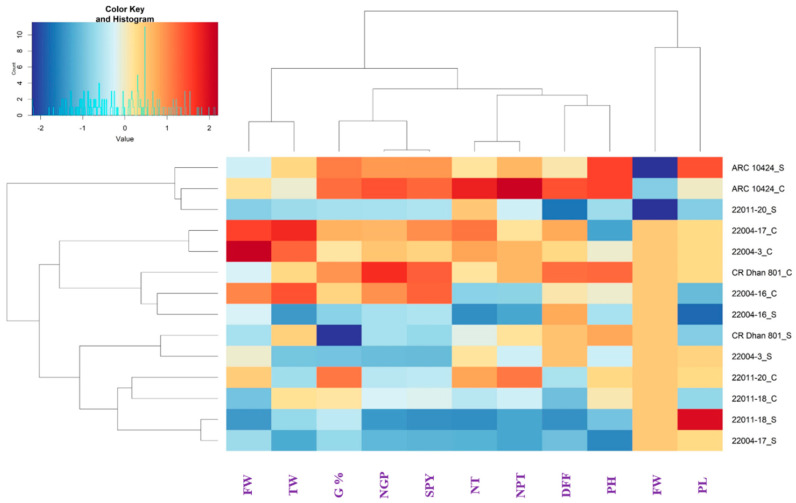
Heatmap and hierarchical clustering of selected BC_3_F_3_ introgressed lines, ARC10424 (donor), and CR Dhan 801 (recipient parents) under control (C) and anaerobic stress (S) conditions. Evaluated traits include flag leaf width (FW), test weight (TW), germination percentage (G%), number of grains per panicle (NGP), single plant yield (SPY), number of tillers (NTs), number of productive tillers (NPTs), days to 50% flowering (DFF), plant height (PH), flag leaf width (FW), and panicle length (PL). Colour gradients represent warmer colours (red/orange), indicating higher values and cooler colours (blue), indicating lower values.

Five introgressed lines containing the QTLs *qAG2.1*, SUB1, *qDTY1.1*, and *qDTY3.1* at the BC_3_F_3_ generation, along with the donor (ARC10424) and recipient (CR Dhan 801) parents, were evaluated for germination under both normal and anaerobic stress conditions, as well as for a range of yield and agro-morphological traits (Table 3). Significant genotypic variation was observed, particularly under anaerobic stress. The recipient parent, CR Dhan 801, exhibited a poor AG% of 17.6%, indicating high susceptibility to oxygen-deficient conditions. In contrast, the donor ARC10424 recorded an AG% of 82.6%, confirming its strong tolerance. Among the introgressed lines, the AG% ranged from 41.5% to 52.3%, with lines 22011-18 (52.3%), 22004-17 (46.5%), and 22004-16 (44.5%) showing significantly improved tolerance and successful introgression of the major QTL *qAG2.1* (Figure 6).

Agro-morphological traits under stress conditions also revealed promising variation. Days to 50% flowering (DFF) ranged from 78 to 89 days, with early flowering observed in lines like 22011-20 (78 days) and 22011-18 (79 days), offering potential earliness advantages in stress-prone environments. However, in the case of the plant height (PH), lines 22004-17 and 22004-3 had an average of 66.1 cm and 76.1 cm, respectively, values that were lower than that of CR Dhan (85.3 cm) but were still at a desirable level agronomically. Flag leaf traits remained stable across genotypes, with 22004-3 showing the longest flag leaf under stress (27.5 cm). Yield-related traits, such as number of grains per panicle (NGP) and productive tillers (NPTs), remained relatively consistent, with line 22011-20, matching CR Dhan 801 in NGP (78 grains). Test weight (TW) under stress ranged from 13.04 g to 15.16 g, and several introgressed lines surpassed the recipient parent in single plant yield (SPY). Lines 22004-16 and 22011-20 recorded the highest SPY under stress (15.68 g), followed by 22004-3 (13.07 g), with all outperforming CR Dhan 801 (14.72 g), indicating improved yield stability under anaerobic conditions. To further elucidate these phenotypic observations, a heatmap with hierarchical clustering was generated using standardised trait data (Figure 6). This multivariate analysis differentiated tolerant and susceptible genotypes, and the tolerant genotype is similar to CR Dhan 801. High-performing lines, such as 22011-18, 22004-16, and 22004-17, clustered closely together, characterised by higher AGMP, SPY, and test weight, reinforcing their consistent performance under stress. The donor ARC10424 also clustered near several improved lines, while CR Dhan 801 grouped separately, reflecting its poor AGMP and overall stress performance.

## 3. Discussion

Rice varieties with the ability to tolerate anaerobic germination are particularly suitable for direct sowing practices. The methodology employed to evaluate this trait involves measuring the germination and emergence of rice seedlings above the water surface after the seeds are sown and subsequently submerged at a depth of 10–20 cm for a duration of 21 days. This approach has facilitated the identification of several QTLs [4]. To achieve a more comprehensive understanding of AG tolerance in rice, this study examined various traits associated with AGT. These traits include the length of the first internode, shoot length, root length, number of leaves, and both shoot and root biomass. Advanced mapping techniques, such as IM and ICIM, were utilised to identify QTLs linked to AGT traits. The F_2:3_ mapping population, derived from the cross between CR Dhan 801 and ARC10424, along with the parent lines, underwent screening for anaerobic germination. ARC10424 exhibited a higher germination percentage, a longer first internode, and increased shoot length compared to CR Dhan 801, indicating superior tolerance to anaerobic conditions in ARC10424 [3,37]. A positive correlation was observed between germination percentage and shoot length, traits that aid in oxygen acquisition from the environment. This observation aligns with the findings reported by [5,38].

### 3.1. Genomic Colocalization Analysis of QTLs with Known Genes

**Figure 7:**
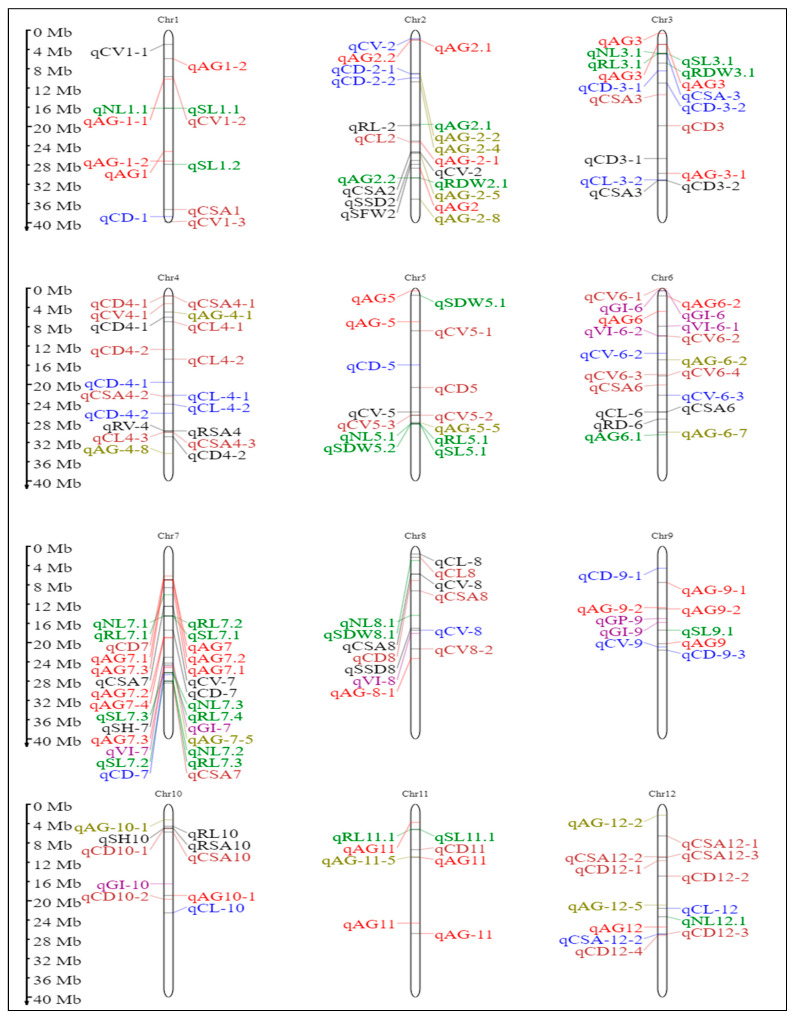
Quantitative trait loci (QTLs) associated with anaerobic germination traits were identified alongside previously reported QTLs linked to various anaerobic germination tolerance traits. QTLs identified in the present study (green) and QTLs identified for anaerobic germination (AG) were represented in (red) USSR5 X N22 [55], IR42/Ma-Zhan Red [7], IR64/Khao Hlan On [40], IR64/Kharsu 80A [56], IR64/Nanhi [8], BJ1/NSIC Rc 222 [57], IR64-AG1 [58], 48 rice genotypes [59], QTLs identified through GWAS studies (Olive) [9], QTLs identified for other anaerobic germination tolerant traits (Purple) YZX/02428 [42], (blue) 02428/YZX [41], (black) H335/CHA-1 [39], and (brown) TD70/Kasalath [60].

A total of 33 QTLs related to various anaerobic germination traits were identified through ICIM. Among these QTLs, some were found to be unique, while others co-localized with previously reported QTLs from different biparental mapping populations (Figure 7). The major QTL identified as a target, *qAG2.1*, was located on chromosome 2 (between 24.42 and 35.37 Mb) and co-localized with several known QTLs, including *qCV-2*, *qCSA2*, *qCD2-1*, *qSSD2*, and *SFW2* [39], as well as *qAG-2-5* and *qAG-2-8* [9]. Additionally, it is situated near *qAG2.1*, which is observed in the IR64/Nanhi population [8]. Another significant region, *qSL1.2*, is found on chromosome 1 and aligns with *qAG-1-2* (from the IR64/Khao Hlan On population) and *qAG1* (from Nampyeong/PBR) [11,40]. A summary of five other QTLs that were earlier reported to be associated with various QTLs is provided in Table 4.

**Table 4:** Summary of QTLs linked to anaerobic germination tolerance and associated traits identified in current and previous studies.

Sl. No	QTL	Chromosome	Trait(s)	Co-Localized QTLs	Reference(s)
1	*qSL3.1*	3	Shoot Length	qNL3.1, qRDW3.1, qRL3.1, qAG3, qCSA-3	[8,41]
2	*qSL5.1*	5	Shoot Length	qNL5.1, qRL5.1, qSDW5.1, qAG-5-5	[9]
3	*qSL9.1*	9	Shoot Length	qGP-9, qGI-9	[42]
4	*qSL11.1*	11	Shoot Length	qRL11.1, qAG11	[43]
5	*qSDW5.1*	5	Shoot Dry Biomass	qAG5	[7]

### 3.2. Functional Characterisation and Exploring Haplotype Variation in Candidate Genes for AGT

The QTL *qAG2.1*, which co-localises with several previously reported QTLs for key traits associated with AGT, was selected for the identification of putatively expressed candidate genes within its genomic region. In silico analyses were performed to explore genes involved in hormonal regulation, stress adaptation, and energy metabolism under anaerobic conditions—critical pathways influencing seed germination and seedling growth in submerged environments. Several hormone-related candidate genes were identified in the *qAG2.1* region, of which ten genes with sequence information in the BAAP and consisting of nsSNPs were selected for further downstream analysis. To investigate the functional significance of these genes, haplotype analyses were conducted using the BAAP evaluated under anaerobic conditions.

#### 3.2.1. Hormonal Regulation of Anaerobic Germination

A key function of ethylene is to regulate the rapid elongation of leaves and stems, a mechanism termed the escape strategy, which is especially prominent in deep-water rice [15]. Central to this process are the *ERF* and *ACO* genes, which are crucial for ethylene biosynthesis [12,13,14]. The gene *OsERF109* has dissected the panel into haplotypes with the wild-type (Nipponbare type) and mutant allele. The haplotype with the Nipponbare allele exhibited significantly lower germination percentages (G%) compared to the haplotype associated with mutant alleles. This observation aligns with the results by Yu et al., 2017 [16], who reported that Nipponbare rice lines, characterized by reduced ethylene production, contrasted with knockdown RNA-interference lines (RI) that exhibited elevated ethylene. In contrast, the *OsACO3* gene, which features seven nsSNPs, grouped the panel into two distinct haplotype groups. Among these, the haplotype carrying the Nipponbare allele sequence exhibited a higher G% compared to the haplotype associated with mutant alleles. This indicates that the mutant lines carrying loss-of-function alleles of *OsACO3* fail to sustain ethylene production at levels required under hypoxic conditions.

Under submergence conditions, increased ethylene levels lead to the downregulation of ABA-biosynthetic genes. Specifically, *OsNCED1* (LOC_Os02g47510) encodes 9-cis-epoxycarotenoid dioxygenase, an enzyme crucial for catalysing the oxidative cleavage of 9-cis-epoxycarotenoids, such as neoxanthin and violaxanthin, to produce xanthoxin, an essential precursor in the biosynthesis of abscisic acid (ABA) in higher plants [17]. In the context of haplotype variations, the haplotype group Hap A, carrying the wild-type sequence, had a lower germination percentage. Conversely, the haplotype with mutated sequences showed a higher G%. This might be due to reduced upregulation of *NCED* genes, resulting in comparatively lower levels of ABA accumulation.

Increased ethylene concentrations have been shown to upregulate various gibberellic acid (GA) biosynthesis genes [44,45]. Notably, the gene *OsGA2ox9* (LOC_Os02g41954), which encodes gibberellin 2-oxidase, plays a critical role in modulating gibberellin, and *OsGA2ox9* modulates gibberellin homeostasis and fine-tunes GA levels during germination [18]. This gene displayed two nsSNPs, and the haplotype analysis identified three distinct haplotype groups (HAP C and HAP A were characterised by mutated SNPs at single positions and showed higher G%, compared to the Nipponbare allele sequence in HAP B). However, no significant difference in G% was observed between the reference and mutated haplotype groups. The allelic variants distinguishing the haplotypes might be causing conservative amino acid substitutions in the protein.

#### 3.2.2. Starch Mobilisation and Energy Metabolism Under Anaerobic Stress

Trehalose 6-phosphate (T6P) is a key signal metabolite linking growth to carbon metabolism and reflects sucrose status [46,47,48]. The role of *OsTPS5* (LOC_Os02g54820) in abscisic acid (ABA) signalling during seed germination in Arabidopsis is crucial [22]. The knockout of *OsTPS5* results in a reduced accumulation of trehalose and other soluble carbohydrates, which are vital energy sources during anaerobic germination. In the second step of trehalose metabolism in plants, several genes resembling those found in rice have been identified, including *OsTPP1* (LOC_Os02g44230), in the conversion of T6P to trehalose [21,49]. The *tpp1* mutant showed no detectable *OsTPP1* transcripts, confirming its classification as a loss-of-function mutant. The higher G% observed in the haplotypes of *OsTPS5* and *OsTPP1* with allele sequences resembling the Nipponbare reference genome, when compared to the mutated haplotypes, is likely attributed to the upregulation of these genes. Conversely, the mutated haplotypes may be harbouring loss-of-function alleles with decreased enzyme activity and trehalose levels, ultimately resulting in reduced germination.

#### 3.2.3. Aldehyde Dehydrogenase-Mediated Detoxification During Anaerobic Germination

The enhanced activity of the aldehyde dehydrogenase (*OsALDH2A*) (LOC_Os02g49720) is particularly effective in detoxifying acetaldehyde, a toxic by-product that accumulates under low oxygen conditions [25,50]. This enzyme’s activity is notably elevated in rice varieties capable of successful germination under anaerobic conditions [25]. Proteomics studies further support this association, revealing significant upregulation of *OsALDH2A* in seeds exposed to anoxic conditions. For instance, Lasanthi-Kudahettige, 2007 [26] found an 11-fold increase in *OsALDH2A* expression, while *OsALDH2B* expression was downregulated by 22-fold under similar circumstances. The authors of [51,52] corroborated these patterns, noting that *OsALDH2A* expression peaks between 12 and 72 h after anoxia begins. This increased enzyme activity correlates with improved germination performance under stress. Consistent with these findings, genotypes possessing the reference haplotype displayed a higher germination percentage (G%) than those with the mutant haplotype, suggesting that the mutant phenotypes have a defective protein or have lost efficient function.

#### 3.2.4. Integrative Functions of Water Channel Proteins and Nitric Oxide Signalling Under Hypoxic Conditions

Nitric oxide (NO) impacts seed germination by decreasing respiration rates, minimising reactive oxygen species (ROS), and enhancing the metabolism of carbohydrates, amino acids, and organic acids. It also amplifies α-amylase activity in seeds, which is vital for their survival. However, sustained exposure to NO can diminish α-amylase activity due to the accumulation of reactive nitrogen species (RNS). Moreover, NO regulates seed dormancy by degrading the *ABI5* protein, facilitating the breakdown of abscisic acid (ABA), and increasing the activity of antioxidant enzymes [53]. Aligning with these findings, the haplotype featuring the Nipponbare allele might be leading to increased levels of NO, resulting in lower germination percentages compared to the mutated haplotype, as seen in this study. NO has been shown to enhance the expression of four out of eleven rice plasma membrane intrinsic proteins (PIPs), which play a vital role in regulating water flow within and between plant cells. These proteins are classified into two groups, PIP1 and PIP2, akin to classifications found in *Arabidopsis thaliana* and *Zea mays*. Among these aquaporins, *OsPIP1;3* stands out for its significant contribution to seed germination. Research reported by Liu et al., 2007 [27] revealed that the expression of *OsPIP1;3* was initially low at 12 h post-germination, increased at 24 h, reached its peak at 48 h, and subsequently decreased by 72 h. The study further indicated that germination rates were notably higher in wild-type and sense-transgenic seeds compared to antisense-transgenic seeds, underscoring the crucial role of *OsPIP1;3* in facilitating efficient seed germination [54]. Consistent with these observations, it is expected that the haplotype containing the Nipponbare allele sequence will exhibit elevated levels of *OsPIP1;3*, potentially resulting in improved germination rates relative to the mutated haplotype.

### 3.3. A Putative Pathway for Hypoxic Seed Germination: Integrating Ethylene and Metabolic Adaptations

**Figure 8:**
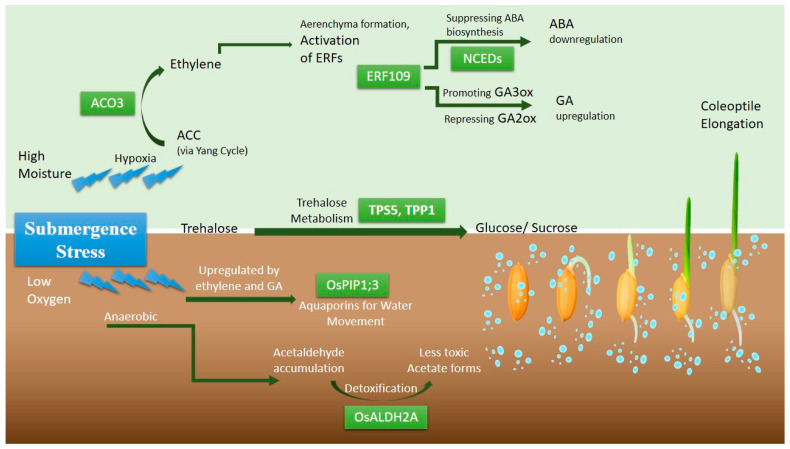
Integrated pathway of ethylene signalling, hormonal regulation, and metabolic adaptation during hypoxic germination. Green rectangles represent key genes identified in the present study, while green arrows indicate upregulation.

Based on genetic association, haplotype differentiation, and the established literature, we propose a working model for hypoxic germination (Figure 8); this represents a hypothesis-generating framework, and causal relationships require experimental validation. The genes identified in this study suggest a pathway for seed germination under hypoxic conditions that integrates key molecular processes involving ethylene signalling, hormonal regulation, starch metabolism, protective mechanisms, and water dynamics regulation. Under anaerobic stress, the upregulation of the *ACO* gene, particularly *OsACO3*, triggers ethylene biosynthesis, which activates ethylene response factors. These ERFs mediate the expression of downstream genes involved in growth elongation and metabolic adaptation. Ethylene also interacts with abscisic acid (ABA), leading to the downregulation of ABA biosynthetic genes (*OsNCED1*, *OsNCED2*, and *OsNCED5*), reducing ABA levels and alleviating dormancy, thus facilitating germination. Ethylene further activates gibberellin (GA) homeostasis modulation, including genes like *OsGA2ox9*, which fine-tune GA levels during germination and indirectly influence starch mobilisation via α-amylase (Osα-Amy), thereby contributing to energy supply under hypoxic conditions. Concurrently, trehalose metabolism, regulated by *OsTPS5* and *OsTPP1*, provides an additional osmotic regulator and energy reservoir, essential for germination. The detoxification of acetaldehyde by the aldehyde dehydrogenase enzyme *OsALDH2A* protects the seed from toxic by-products of anaerobic metabolism. Water uptake is facilitated by aquaporins such as *OsPIP1;3*, whose expression is enhanced by nitric oxide (NO) signalling, promoting hydration and accelerating germination. NO also boosts starch hydrolysis by activating α-amylase and regulating carbohydrate metabolism, leading to increased glucose and sucrose concentrations necessary for seedling growth. Together, these interconnected processes support seed germination under hypoxic conditions by optimising energy metabolism, cellular protection, and hydration.

### 3.4. Assessing the Impact of Crop Establishment, Growth and Yield Under Normal and Anaerobic Conditions

The evaluation of BC_3_F_3_ introgressed lines for anaerobic germination (AG) tolerance and related agro-morphological traits under both normal and stress conditions revealed substantial genetic variability, affirming the effectiveness of marker-assisted introgression of major QTLs (*qAG2.1*, *Sub1*, *qDTY1.1*, and *qDTY3.1*). Notably, lines such as 22011-18, 22004-17, and 22004-16 displayed enhanced anaerobic germination percentage, ranging from 44.5% to 52.3%, significantly outperforming the susceptible recipient parent CR Dhan 801 (17.6%). These observations suggest the successful incorporation and functioning of *qAG2.1*, a key QTL known to regulate anaerobic germination by enhancing coleoptile elongation and seedling survival under low oxygen conditions. The heatmap and hierarchical clustering analysis (Figure 6) provided further insights into the phenotypic relationships among the genotypes. The clustering distinctly separated the donor ARC10424 and several high-performing introgressed lines (e.g., 22011-18, 22004-16, and 22004-17) from the susceptible parent CR Dhan 801. These tolerant lines formed a cluster characterised by higher AG%, greater single plant yield, and relatively stable morphological traits under stress. This clustering pattern supports the phenotypic data and emphasises the consistency and reliability of the selected traits in discriminating AG-tolerant lines.

Interestingly, some introgressed lines (e.g., 22011-20 and 22004-3) not only maintained better AG% and yield under stress but also demonstrated early flowering and AG-adapted plant height, traits that are particularly beneficial in direct-seeded rice (DSR) [24]. The early flowering observed in these lines may allow them to escape late-season stresses and could be advantageous in synchronising maturity with local climatic patterns. While most lines showed a decrease in productive tillers and grains per panicle under stress, the best-performing lines sustained values at par or exceeding those of CR Dhan 801. The stability of test weight across stress and normal conditions further suggests that grain filling processes in these lines were not significantly disrupted under anaerobic conditions. This is crucial, as yield stability under stress conditions is a major breeding goal. Moreover, the clustering of CR Dhan 801, apart from tolerant lines in the heatmap, reinforces its poor performance and susceptibility to anaerobic stress, while ARC10424, as expected, aligned closely with several improved lines, indicating that key QTLs from the donor were effectively transferred and expressed in the progeny. The combined phenotypic and multivariate analysis confirms that several BC_3_F_3_ lines exhibit not only improved anaerobic germination but also maintain agronomic performance and yield stability under stress. These findings highlight the potential of lines such as 22011-18, 22004-16, and 22011-20 as promising candidates for further advancement and release in breeding programs targeting direct-seeded and flood-prone environments. Collectively, the combined trait analysis and clustering approach confirm the potential of these introgressed lines for deployment in direct-seeded and flood-prone rice ecosystems. While the present study provides robust genetic and breeding-level validation of *qAG2.1*, direct functional validation of the underlying putative genes was beyond the scope of this work and remains an important priority for future studies.

## 4. Materials and Methods

### 4.1. Plant Material and Germplasm

**Figure 9:**
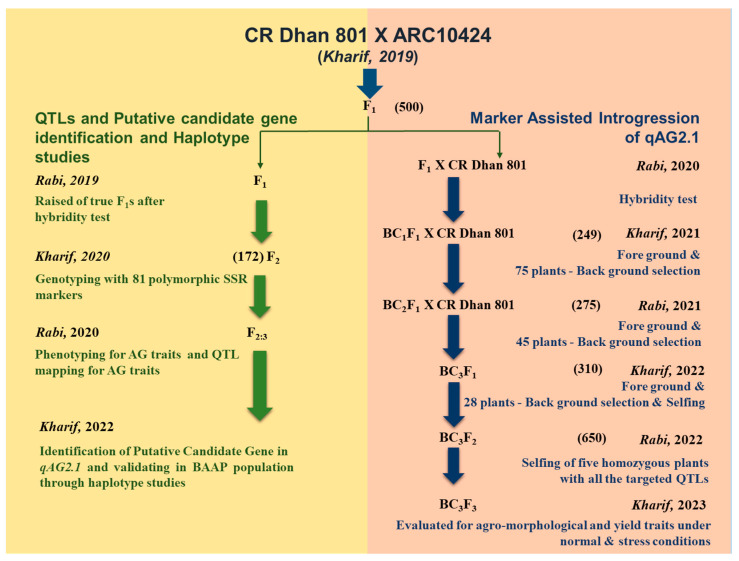
Schematic flow of work for identification of QTLs, introgression of *qAG2.1* into variety CR Dhan 801 through MAS (figure in parentheses indicates the number of hybrids/lines raised in the respective generation), and candidate gene identification followed by haplotype studies.

The experimental population comprising 172 F_2:3_ progenies was developed by crossing CR Dhan 801 with ARC10424 at the ICAR-National Rice Research Institute (CRRI), Cuttack, located at 20.5° N, 86° E, and 23.5m above mean sea level. CR Dhan 801, a cultivar bred by ICAR-CRRI, harbours the *Sub1* gene, imparting submergence tolerance, along with stacked *qDTY1.1*, *qDTY2.1*, and *qDTY3.1* QTLs, imparting tolerance to drought stress. While exhibiting submergence tolerance during the vegetative phase for 12–14 days, CR Dhan 801 lacks the requisite resilience during germination. Numerous germplasms globally have been identified as potential sources for AGT. Within the confines of ICAR-CRRI, landraces such as China Mali, Panirohi, BJ1, and ARC10424 have been ascertained as promising donors for this trait [61]. Consequently, ARC10424 was chosen as the donor for AGT and, using CR Dhan 801 and ARC10424 as parental genotypes, the mapping population was generated to identify AGT QTLs. Figure 9 represents the schematic diagram of the workflow used in the current study.

### 4.2. Genotyping of Mapping Population

The mapping population of 172 F_2_ lines was genotyped to identify QTLs. The DNA was extracted from leaf samples at the tillering stage via the modified miniprep CTAB method [62]. DNA was quantified to 50 ng/μL using a nano-drop spectrophotometer and checked for quality on a 1.2% agarose gel. A survey of parental polymorphism was performed between CR Dhan 801 and ARC10424, utilising 702 Simple Sequence Repeat (SSR) markers. From this analysis, 81 polymorphic markers were selected for QTL mapping (Supplementary Table S6). The SSR marker sequences and related details were sourced from the Gramene markers database (http://www.gramene.org.in), and genotyping was carried out using the selected polymorphic SSR markers. PCR amplification was done in a 10 μL reaction with 2 μL DNA template, 4 μL Master mix, and 4 μL MilliQ water, following a specific amplification profile. PCR products, mixed with bromophenol blue dye, were run on 4.5% agarose gels with a 1 kb+ ladder for 2 h.

### 4.3. Phenotypic Screening for AGT in Mapping Population

In the 2022 dry (*Rabi*) season, 172 F_2:3_ lines were screened in the experimental setup (a seedling tray containing 156 wells arranged in a 13-column by 12-row configuration). Each F_2:3_ line was represented by 72 healthy dry seeds evenly distributed across 12 wells. These wells were filled with a 3.5 cm layer of fine soil beneath the seeds, covered by a 1 cm layer of well-dried field soil above the seeds. For comparative analysis, both the donor-tolerant parent ARC10424 and the susceptible recurrent parent, CR Dhan 801, were included in each of the trays as positive and negative controls, respectively. Following seeding, the seedling trays were carefully placed in a larger container filled with water, ensuring a consistent depth of 10 cm above the tray throughout the 21-day experimental period. A random selection of twelve seedlings from each genotype was phenotyped at 21 days after sowing (DAS). Phenotyping and subsequent analysis were carried out for all germinated entries, with entries failing to germinate being excluded from the analysis of seedling morphological traits. Prior to phenotyping, uprooted seedlings were carefully rinsed with running tap water to remove soil residue. The anaerobic germination percentage was determined by assessing the number of plants that emerged above the water surface relative to the total number of seeds sown. For morphological measurements, shoot length was determined from the shoot’s base to its tip, while the first internode length was calculated from the base of the shoot to the first node. Root length was measured from the crown root base to the tip of the longest root. Subsequently, the seedlings were adequately dried in an air-forced oven at 60 °C for 5–6 days and weighed to calculate shoot and root biomass in grams.

### 4.4. Linkage Map Construction and Identification of QTLs for AGT Traits

The linkage between genetic markers and quantitative trait loci (QTL) was evaluated utilising the logarithm of odds (LODs) score method. A set of 704 SSR markers, with map positions obtained from the Gramene database (www.gramene.org) were assessed for polymorphism. The polymorphic markers (81) were grouped into 12 linkage groups utilising anchor information and establishing an anchor order for optimal marker positioning. The integrated software QTL IciMapping V.4 [63] facilitated the detection of QTLs through interval mapping (IM) and inclusive composite interval mapping for additive QTL (ICIM-ADD) methodologies. The construction of linkage maps was achieved utilising the MAP function within the biparental population module of the ICIM software, employing the Kosambi mapping function.

### 4.5. Identification of Putative Candidate Genes Within the QTL Region Superior Haplotypes and Potential Donors in the BAAP Panel

The length and positions of the significant QTL, *qAG2.1,* were detected utilising the knowledge of flanking markers from the Gramene database. The functional annotation of the genes present in this region was done using Rice Genome Annotation Project (RGAP) version 7. Priority was given to genes associated with functions pertinent to anaerobic germination. Haplotype analysis was performed by examining non-synonymous single-nucleotide polymorphisms (nsSNPs) within exon regions of the Nipponbare reference genome, utilising the sequence information of BAAP in the SNP-Seek database. These nsSNPs facilitated the stratification of the population into haplotypes, highlighting the effect of putative genes via the haplotype means of AG%. The BAAP was phenotyped for AGT traits and was reported in our previous work [3].

### 4.6. Introgression of AGT QTL and Evaluation of MABC-Derived Lines Under Normal and Stress Conditions

The major QTL, *qAG2.1,* detected in the CR Dhan 801/ARC10424 mapping population (F_2:3_), was introgressed into CR Dhan 801 using a marker-assisted backcross breeding (MABB) scheme. The introgression process began with the hybridisation of CR Dhan 801 with ARC10424, followed by backcrossing the F_1_ progeny to CR Dhan 801. A total of 500 F_1_ seeds were produced by crossing CR Dhan 801 with ARC10424, and the true F_1_ plants were crossed with the recipient parent; CR Dhan 801 and 424 BC_1_F_1_ seeds were generated. Of which 300 seeds were used to raise the BC_1_F_1_ generation (the rest of the seeds were stored as a backup). However, only 249 of them survived in BC_1_F_1_. In each generation, approximately 250–300 plants were genotyped to verify the presence of the target QTL, and only those with the target QTL were advanced to the next generation. Foreground selection was done using the flanking markers RM207 and RM6318, while the 81 polymorphic markers identified in this study were used for background genome selection. In each generation, approximately 250–300 plants were genotyped to verify the presence of the target QTL. The genomic selection continued until the BC_3_F_2_ generation. In BC_3_F_2_, the homozygous lines for *qAG2.1* were subsequently evaluated for the presence of Sub1, *qDTY1.1*, *qDTY2.1*, and *qDTY3.1*.

A set of five BC_3_F_3_ pyramided lines, along with their parents, were subjected to anaerobic stress during germination for 21 days, followed by a 10-day recovery period. A second set of these plants was also allowed to germinate under normal conditions. Germination percentages were recorded for both stress-treated and control conditions. Thirty-one-day-old seedlings from both experiments were then transplanted in a randomised complete block design with two replications. The planting arrangement consisted of three rows per entry, with twenty plants per row and a spacing of 20 × 15 cm, and standard agronomic practices were followed. Data were collected at grain maturity stage, ten plants per entry in each replication were sampled for the following agronomic traits: flag leaf length (FL), flag leaf width (FW), plant height (PH), number of tillers per plant (NT), number of productive tillers per plant (NPT), panicle length (PL), number of grains per panicle (NGP), 1000-grain weight (TW), and single plant yield (SPY). Additionally, days to 50% flowering (DFF) were recorded for the entire plot.

### 4.7. Statistical Analysis and Graphical Illustrations

Descriptive statistics were calculated for the phenotypic traits of the mapping population using GRAPES [64] and correlation using the R package corrplot [65]. The ridgeline plot to illustrate the haplotype distribution for various genes was plotted using the Lineplot option from the SRplot bioinformatics web tool [66]. A GGT plot was created to illustrate the genome recovery percentage of the selected five lines positive for the target QTLs using GGT software 2.0 [67]. Heatmap and hierarchical clustering of selected BC_3_F_3_ introgressed lines were performed using the ComplexHeatmap package in R [68]. Prior to heatmap generation, trait data were standardised using Z-score normalisation, and treatments were clustered using Ward’s minimum variance method (ward.D2) based on Euclidean distance, while trait order was retained to aid physiological interpretation. The significance of the difference between the haplotype means was calculated using One-Way ANOVA, and the Games–Howell test (assuming unequal variance) was used for post-hoc analysis using Minitab v 19.1 (Minitab Inc., State College, PA, USA). The chromosome map illustrated all the identified QTLs associated with anaerobic germination traits identified, alongside previously reported QTLs linked to various AGT traits, using Mapgene2chrom web tool [69].

## 5. Conclusions

This study successfully dissected the genetic architecture of AGT in rice through QTL mapping in an F_2:3_ population derived from CR Dhan 801 × ARC10424. A major QTL, *qAG2.1*, located on chromosome 2, was consistently associated with key AGT-related traits and co-localised with previously reported QTLs. In silico analysis of the *qAG2.1* region revealed several candidate genes involved in crucial pathways, such as ethylene biosynthesis (*OsERF109*, *OsACO3*), ABA signalling (*OsNCED1*), gibberellin metabolism (*OsGA2ox9*), carbohydrate mobilisation (*OsTPS5*, *OsTPP1*), aldehyde detoxification (*OsALDH2A*), and water/nitric oxide signalling (*OsPIP1;3*). Haplotype analysis across a diverse panel confirmed that allelic variations in these genes significantly influenced germination efficiency under hypoxic conditions, supporting their functional role in AGT. To translate these findings into breeding applications, *qAG2.1* was introgressed into the high-yielding, stress-tolerant variety CR Dhan 801, which already carries SUB1, qDTY1.1, and qDTY3.1 QTLs. Phenotypic assessment in BC_3_F_3_ generation revealed that lines such as 22011-18, 22004-16, and 22011-20 exhibited higher anaerobic germination percentages (up to 52.3%), along with early vigour, improved yield, and stable agronomic traits under stress, without compromising performance under normal conditions. This study extends the stress-resilience portfolio of the multi-stress-tolerant rice variety CR Dhan 801 by introgressing the anaerobic germination tolerance locus *qAG2.1*, thereby enhancing seedling establishment under flooded direct-seeded conditions without compromising agronomic performance. Together, these results elevate *qAG2.1* from a mapped locus to a practical breeding target and a testable regulatory hub for hypoxic germination.

## Figures and Tables

**Table 1:** Descriptive statistics of various anaerobic germination-related traits of F_2:3_ mapping population phenotyped under anaerobic stress (CD—Critical Difference, SEM—Standard Error of mean, CV—Coefficient of Variation, GCV—Genotypic Coefficient of Variation, PCV—Phenotypic Coefficient of Variation).

Characters	Range	Mean	CD (5%)	SEM	CV	GCV	PCV	Skewness	Kurtosis
Germination percentage (%)	0–94.44	38.33	4.8	1.72	7.8	69.3	72.5	0.31	−1.13
Shoot length (cm)	14.4–34.41	25.99	2.3	0.82	5.6	22.0	24.1	−2.18	7.82
Root length (cm)	3.5–18.50	9.76	1.18	0.42	7.7	25.4	28.3	−0.97	4.96
Length of first internode (cm)	1.19–8.89	5.1	0.3	0.1	3.7	30.5	31.5	0.02	2.19
Number of leaves	2–3.7	2.88	0.1	0.03	6.7	19.7	20.8	−0.05	0.47
Shoot dry biomass (g)	0.002–0.140	0.081	0.006	0.002	4.3	48.6	49.4	−0.67	−0.78
Root dry biomass (g)	0.002–0.079	0.04	0.005	0.002	6.6	74.8	76.7	−0.16	−1.3

**Table 2:** QTLs detected for traits related to anaerobic germination tolerance in the F_2:3_ lines by IM and ICIM methods.

Sl. No	QTL	Chromosome	Markers	LOD		PVE (%)		ADD		Dom		IAE	
				IM	ICIM	IM	ICIM	IM	ICIM	IM	ICIM	IM	ICIM
1	*qSL1.1*	1	RM259–RM9	12	12	2.15	2.15	13.58	13.58	11.57	11.57	C	C
2	*qSL1.2*	1	RM9–RM128	9	9	1.62	1.62	13.69	13.69	11.72	11.72	C	C
3	*qNL1.1*	1	RM259–RM9	21.41	21.41	1.23	1.23	1.52	1.52	1.44	1.44	C	C
4	*qAG2.1*	2	RM207–RM6318	2.85	3.06	12.4	5.45	5.23	4.56	3.08	4.38	C	C
5	*qAG2.2*	2	RM279–RM207	-	3.26	-	11.69	-	−1.01	-	−11.6	x	A
6	*qIL2.1*	2	RM324–RM263	3.38	-	7.58	-	−1.21	-	−1.35	-	A	x
7	*qSDW2.1*	2	RM324–RM263	3.35	-	7.9	-	0.01	-	0.17	-	C	x
8	*qSDW2.2*	2	RM279–RM207	3.84	-	7.97	-	−0.03	-	−0.18	-	A	x
9	*qRDW2.1*	2	RM207–RM6318	3.96	3.96	3.07	3.07	0.03	0.03	−0.03	−0.03	C	C
10	*qSL3.1*	3	RM218–RM3894	4.48	4.48	1.94	1.94	9.31	9.31	10.11	10.11	C	C
11	*qRL3.1*	3	RM218–RM3894	2.59	2.59	1.89	1.89	2.44	2.44	2.87	2.87	C	C
12	*qNL3.1*	3	RM218–RM3894	13.66	13.66	1.28	1.28	1.41	1.41	1.65	1.65	C	C
13	*qRDW3.1*	3	RM3894–RM282	4.79	3	4.12	7.56	−0.04	−0.03	−0.04	−0.04	A	A
14	*qSL5.1*	5	RM30–RM334	16.71	16.71	2.04	2.04	−13.15	−13.15	12.77	12.77	A	A
15	*qRL5.1*	5	RM30–RM334	8.26	8.26	2.49	2.49	−3.82	−3.82	5.45	5.45	A	A
16	*qNL5.1*	5	RM30–RM334	23.9	23.9	1.24	1.24	−1.52	−1.52	1.37	1.37	A	A
17	*qSDW5.1*	5	RM2010–RM17847	4.78	4.78	3.6	3.6	−0.01	−0.01	0.17	0.17	A	A
18	*qSDW5.2*	5	RM2010–RM17847	4.99	4.99	3.89	3.89	−0.05	−0.05	−0.16	−0.16	A	A
19	*qSDW5.3*	5	RM30–RM334	4.32	4.32	3.58	3.58	0.01	0.01	0.18	0.18	C	C
20	*qRDW5.1*	5	RM2010–RM17847	4.62	-	5.22	-	−0.04	-	−0.04	-	A	x
21	*qRDW5.2*	5	RM30–RM334	3.24	-	4.51	-	0.01	-	0.06	-	C	x
22	*qAG6.1*	6	RM7555–RM439	2.67	3.28	3.4	1.67	2.67	2.81	2.84	2.91	C	C
23	*qSL7.1*	7	RM427–RN234	11.41	11.41	1.29	1.29	12.55	12.55	14.36	14.36	C	C
24	*qSL7.2*	7	RM234–RM248	14.05	14.05	1.24	1.24	12.9	12.9	13.52	13.52	C	C
25	*qSL7.3*	7	RM248–RM336	11.17	11.17	1.23	1.23	13.02	13.02	13.16	13.16	C	C
26	*qRL7.1*	7	RM6767–RM427	4.43	3.23	0	0	2.45	2.23	2.84	2.61	C	C
27	*qRL7.2*	7	RM427–RM234	7.4	6.39	0	0	3.94	3.88	4.31	4.08	C	C
28	*qRL7.3*	7	RM234–RM248	9.39	8.47	0	0	4.08	4.02	4.47	4.21	C	C
29	*qRL7.4*	7	RM248–RM336	5.26	4.48	0	0	4.18	4.17	4.55	4.32	C	C
30	*qIL7.1*	7	RM6728–RM11	2.87	-	2.15	-	−0.76	-	−0.64	-	A	x
31	*qNL7.1*	7	RM427–RM234	17.86	14.54	2.45	3.59	1.47	1.36	1.55	1.44	C	C
32	*qNL7.2*	7	RM234–RM248	12.59	9.8	1.97	2.82	1.45	1.31	1.57	1.45	C	C
33	*qNL7.3*	7	RM248–RM336	9.68	6.93	1.96	2.81	1.45	1.31	1.55	1.44	C	C
34	*qNL8.1*	8	RM6925–RM515	7.67	7.67	1.22	1.22	1.48	1.48	1.52	1.52	C	C
35	*qSDW8.1*	8	RM1235–RM1111	2.58	2.58	0.6	0.6	−0.05	−0.05	0.01	0.01	A	A
36	*qSL9.1*	9	RM566–RM242	4.88	4.88	1.63	1.63	−13.36	−13.36	11.43	11.43	A	A
37	*qSL11.1*	11	RM26073–RM3701	2.77	2.77	0.47	0.47	1.75	1.75	2.94	2.94	C	C
38	*qRL11.1*	11	RM26073–RM3701	3.38	3.38	0.81	0.81	1.02	1.02	1.55	1.55	C	C
39	*qNL12.1*	12	RM28759–RM519	13.53	13.53	1.23	1.23	1.52	1.52	1.43	1.43	C	C

Note: AG: anaerobic germination, IL: length of first internode, SL: shoot length, RL: root length, NL: number of leaves, SDW: shoot dry weight, RDW: root dry weight, LOD: logarithm of odds, PVE: phenotypic variance explained, Add: additive, Dom: dominance, IAE: increasing allele effect, C: CR Dhan 801, A: ARC10424, IM: interval mapping, ICIM: inclusive composite interval mapping.

**Table 3:** Agro-morphological and yield characters of five BC_3_F_3_ anaerobic germination-tolerant lines under normal (N) and stress (S) conditions.

Sl. No.	Name of the Entry	NG (%)	AG (%)	DFF N	DFF S	PH (cm) N	PH (cm) S	FL (cm) N	FL (cm) S	FW (cm) N	FW (cm)	NT N	NT S	PL (cm) N	PL (cm) S	NGP N	NGP S	NPT N	NPT S	TW (gm) N	TW (gm) S	SPY (gm) N	SPY (gm) S
P 1	CR Dhan 801	79.7	17.6	91	88	89.1	85.3	26.5	24.7	1.1	1.0	13	12	24.2	22.3	148	78	12	11	14.43	14.69	25.22	14.72
P 2	ARC10424	85.3	82.6	92	86	91.5	91.6	28.7	26.2	0.9	0.8	18	13	23.7	26	139	123	15	12	16.25	16.46	24.85	23.01
1	22004-3	79	41.5	87	88	78.4	76.1	38.3	27.5	1.1	0.9	15	13	24.2	24.3	112	65	12	10	14.83	14.41	20.29	13.07
2	22004-16	68	44.5	86	89	78.4	73.9	32.9	26.5	0.9	1.1	10	8	22	20.8	125	78	9	8	16.8	13.04	25.05	15.68
3	22004-17	75	46.5	89	81	68.1	66.1	35.4	24.2	0.9	1.1	16	9	24.2	24.2	116	63	11	8	15.2	13.49	23.38	12.66
4	22011-18	64	52.3	81	79	79.4	75.8	22.9	20.7	1.0	1.0	13	12	25.5	26.9	89	55	10	8	15.2	15.16	17.47	11.06
5	22011-20	83.7	48.3	83	78	81.5	73.2	29.9	23.5	1.1	0.8	15	14	24.2	22.3	82	78	13	10	15.41	14.25	16.23	15.68
		76.4	47.6	87	84	80.9	77.4	30.7	24.8	1.0	0.9	14	12	24.0	24.0	116	77	12	10	15.4	14.5	21.8	15.1
	CD (5%)	5.87	2.67	6.05	4.89	7.18	5.52	2.73	2.17	0.16	0.1	2.21	1.14	3.02	2.08	11.86	7.79	2.08	1.15	1.85	1.41	3.85	2.01
	CV (%)	4.43	4.49	4.29	3.62	5.16	4.14	5.41	4.85	8.89	6.25	9.98	6.99	7.65	5.22	5.4	4.5	10.9	7.79	6.62	5.07	9.33	5.86

NG%: normal germination percentage, AG%: anaerobic germination percentage, DFF: days to 50% flowering, PH: plant height (cm), FL: flag leaf length (cm), FW: flag leaf width (cm), NT: number of tillers, PL: panicle length (cm), NGP: number of grains per panicle, NPT: number of productive tillers, TW: test weight (gm), SPY: single plant yield (gm), N: normal condition, S: anaerobic germination stress.

## Data Availability

The original contributions presented in the study are included in the article/Supplementary Material. Further inquiries can be directed to the corresponding authors.

## Supplementary Materials

The following supporting information can be downloaded at: https://www.mdpi.com/article/10.3390/plants15050821/s1.
