# Supplementary material for: qAG2.1 Is Associated with Anaerobic Germination Tolerance in Rice Seeds: Evidence from Haplotype Analysis and Marker-Assisted Breeding
Source: Plants (Basel). 2026 Mar 7;15(5):821. doi: 10.3390/plants15050821 (PMC12986987; doi:10.3390/plants15050821)
Supplement: Supplementary file 1 [file plants-15-00821-s001.zip › plants-4137965-supplementary.pdf]

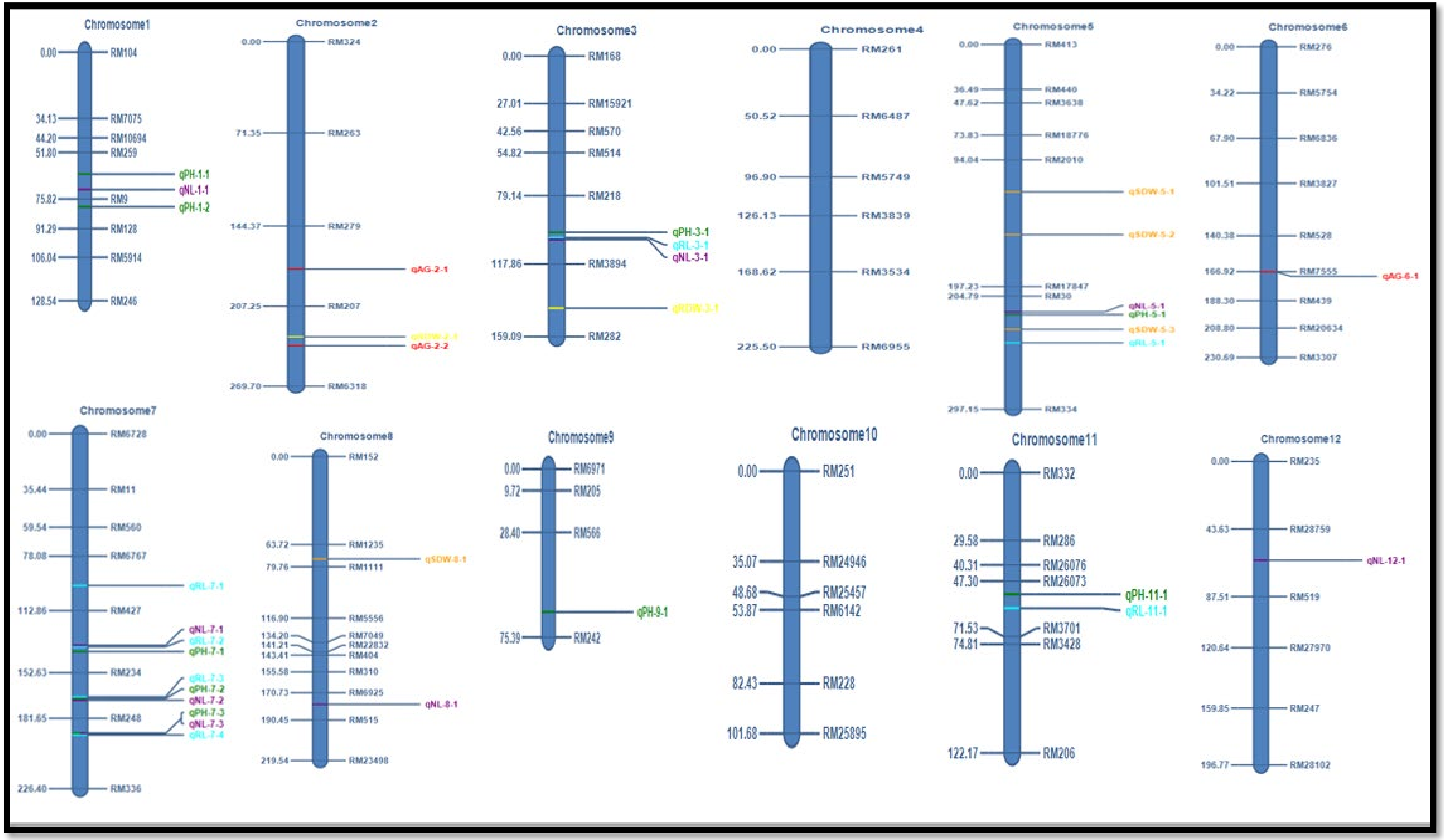

Supplementary Figure 1. Genetic linkage map showing the positions of QTLs for seven traits related to anaerobic germination tolerance in the F2:3 population. QTLs were detected by Inclusive Composite Interval mapping (ICIM) using QTL IciMapping software V. 4.1.
